# Randomized, double-blind, placebo-controlled trial of the once-daily GLP-1 receptor agonist lixisenatide in Asian patients with type 2 diabetes insufficiently controlled on basal insulin with or without a sulfonylurea (GetGoal-L-Asia)*

**DOI:** 10.1111/j.1463-1326.2012.01618.x

**Published:** 2012-05-30

**Authors:** Y Seino, K W Min, E Niemoeller, A Takami, on behalf of the EFC10887 GETGOAL-L Asia Study Investigators

**Affiliations:** 1 Kansai Electric Power HospitalOsaka, Japan; 2 Eulji General HospitalSeoul, South Korea; 3 Sanofi R&DFrankfurt, Germany; 4 Sanofi-aventis K.K.Tokyo, Japan

**Keywords:** Asian patients, basal insulin, GLP-1, lixisenatide, type 2 diabetes

## Abstract

**Aims:**

To assess the efficacy and safety of once-daily lixisenatide versus placebo in Asian patients with type 2 diabetes insufficiently controlled on basal insulin ± sulfonylurea.

**Methods:**

In this 24-week, randomized, double-blind, placebo-controlled, parallel-group, multicentre study, participants (mean baseline HbA_1c_ 8.53%) from Japan, Republic of Korea, Taiwan and the Philippines received lixisenatide (n = 154) or placebo (n = 157) in a stepwise dose increase to 20 µg once daily. The primary endpoint was HbA_1c_ change from baseline to week 24.

**Results:**

Once-daily lixisenatide significantly improved HbA_1c_ versus placebo (LS mean difference vs. placebo = −0.88% [95%CI= −1.116, −0.650]; p < 0.0001), and allowed more patients to achieve HbA_1c_ <7.0% (35.6 vs. 5.2%) and ≤6.5% (17.8 vs. 1.3%). Lixisenatide also significantly improved 2-h postprandial plasma glucose and glucose excursion, average 7-point self-monitored blood glucose and fasting plasma glucose. Lixisenatide was well tolerated; 86% of patients on lixisenatide completed the study versus 92% on placebo. Ten (6.5%) lixisenatide and 9 (5.7%) placebo patients experienced serious adverse events. More lixisenatide patients [14 (9.1%)] discontinued for adverse events versus placebo [5 (3.2%)], mainly with gastrointestinal causes. Nausea and vomiting were reported in 39.6 and 18.2% of patients on lixisenatide versus 4.5 and 1.9% on placebo. Symptomatic hypoglycaemia was more frequent with lixisenatide (42.9%) versus placebo (23.6%), but was similar between groups (32.6 vs. 28.3%, respectively), in those not receiving sulfonylureas. No severe hypoglycaemia was reported.

**Conclusions:**

In an Asian type 2 diabetes population insufficiently controlled by basal insulin ± sulfonylurea, once-daily lixisenatide significantly improved glycaemic control, with a pronounced postprandial effect, and was well tolerated.

## Introduction

Effective treatment of type 2 diabetes requires a multidisciplinary approach, including both lifestyle and pharmacological interventions. Treatment to maintain glycaemic control typically progresses in a stepwise fashion, culminating in the use of multiple oral glucose-lowering agents and/or insulin [1]. Patients with type 2 diabetes exhibit multiple pathophysiological deficits, including declining ß-cell function and a failure to suppress postprandial glucagon secretion [2]. Because of the progressive nature of the disease, currently available glucose-lowering therapies may not control glycaemia adequately in the long term. Optimal drug therapy may also be limited by side effects, such as hypoglycaemia, body weight gain and oedema. Glucagon-like peptide-1 (GLP-1) receptor agonists, such as exenatide and liraglutide, which are incretin hormones, have become established as an important therapeutic option in the management of patients with type 2 diabetes [1,3]. This class of drugs has several advantageous characteristics, including a low propensity to cause hypoglycaemia and the ability to promote weight loss [4,5].

Incretin-based therapies appear to be particularly effective in Asian and Japanese patients with type 2 diabetes (who tend to have a pathophysiology of insulin deficiency rather than insulin resistance), and there is some evidence to suggest a profound underlying GLP-1 insufficiency in these patients [6–8].

Lixisenatide is a new potent and selective once-daily GLP-1 receptor agonist in development for the treatment of type 2 diabetes [9–13]. A 13-week, dose-ranging, Phase II study found the optimal dose of lixisenatide to be 20 µg once daily, with significant improvements in HbA_1c_ versus placebo and a good efficacy/tolerability ratio [11]. Lixisenatide has demonstrated dose-dependent improvements in post-meal glucose levels and suppression of postprandial glucagon secretion in patients with type 2 diabetes insufficiently controlled with metformin, as well as pharmacodynamic effects consistent with a glucose-dependent effect on insulin secretion and suggested improvements in ß-cell function [12].

Several studies have looked at the efficacy and safety of other GLP-1 receptor agonists in Japanese patients or other Asian populations [14–23]; however, these were either as monotherapy or add-on to oral agents and only one GLP-1 study to date has included patients on insulin therapy and only 3% of the total population was Asian [24]. We present the results of a study that assessed the effects on glycaemic control of lixisenatide in comparison to placebo as an add-on treatment to basal insulin with or without sulfonylurea in terms of HbA_1c_ reduction over a period of 24 weeks in Asian patients with type 2 diabetes.

## Material and Methods

### Patients

Male and female patients aged 25–81 years with type 2 diabetes (≥1 year duration) currently on stable basal insulin therapy with or without a sulfonylurea and with HbA_1c_ between 7 and 10%, inclusive, were included in the study. Patients had received treatment with a stable basal insulin regimen for at least 3 months, including a stable (±20%) dose of at least 10 U/day for at least 2 months prior to the screening visit, with or without sulfonylurea at a stable dose for at least 3 months prior to the screening visit.

The main exclusion criteria were: use of oral or injectable glucose-lowering agents other than sulfonylurea or basal insulin within 3 months prior to the time of screening; fasting plasma glucose (FPG) at screening >250 mg/dl (13.9 mmol/l) in order to exclude, in a placebo-controlled study, patients in a severely uncontrolled glycaemic situation; history of unexplained pancreatitis, chronic pancreatitis, pancreatectomy, stomach/gastric surgery or inflammatory bowel disease; history of metabolic acidosis, including diabetic ketoacidosis, within 1 year prior to screening; history within the previous 6 months of myocardial infarction, stroke or heart failure requiring hospitalization or drug or alcohol abuse; uncontrolled/inadequately controlled hypertension at the time of screening, with a resting systolic blood pressure greater than 180 mmHg or diastolic blood pressure greater than 95 mmHg; amylase and/or lipase greater than three times or aspartate aminotransferase (AST), alanine aminotransferase (ALT) or alkaline phosphatase (ALP) greater than two times the upper limit of the normal laboratory range; end-stage renal disease and/or dialysis and clinically relevant history of gastrointestinal disease, with prolonged nausea and vomiting during the previous 6 months.

The study was approved by the institutional review boards or ethics committees and was conducted in accordance with the Declaration of Helsinki and Good Clinical Practice guidelines. All patients gave written informed consent prior to participation in the study.

### Study Design

This was a 24-week, randomized, double-blind, placebo-controlled, two-arm, parallel-group study. It was conducted in 57 centres in four countries in Asia (Japan, Republic of Korea, Taiwan and the Philippines). Following a 2-week screening phase and a 1-week placebo run-in period, eligible patients were randomized in a 1 : 1 ratio to receive lixisenatide (10 µg for 1 week, 15 µg for 1 week, then 20 µg), or placebo, all administered subcutaneously once daily within 1 h before breakfast. The study was double-blind to assigned treatment, but not to treatment volume.

All patients continued treatment throughout the study with their established doses of basal insulin with or without sulfonylureas. In case of screening HbA_1c_ ≤7.5%, the insulin dose was reduced by 20%, otherwise the insulin dose was to be kept stable within ±20% of the screening dose and dose decreases were allowed in the case of two symptomatic or one severe hypoglycaemic event; increases of >20% for >7 days were considered as rescue therapy. In case of screening HbA_1c_ ≤8.0%, the sulfonylurea dose was decreased by ≥25% (or stopped in case of minimum dose) at randomization in order to decrease the risk of hypoglycaemia. Routine fasting self-monitoring plasma glucose (SMPG) and central laboratory alerts on FPG and HbA_1c_ ensured that glycaemic parameters remained under predefined thresholds values. Dietary and lifestyle counselling consistent with international or local guidelines was given to all patients at baseline and week 12.

Randomization of subjects and allocation of medication was performed using an interactive voice response system (IVRS). Patients were stratified by screening values of HbA_1c_ (<8.0%, ≥8.0%) and sulfonylurea use (yes/no). A Data Monitoring Committee (DMC) supervised the conduct of the study by an ongoing review of unblinded safety and main efficacy parameters. An Allergic Reaction Assessment Committee (ARAC) reviewed and adjudicated possibly allergic events in a blinded manner.

### Endpoints and Assessments

The primary efficacy endpoint was change in HbA_1c_ from baseline to week 24 for the modified intent-to-treat (mITT) population, which included all patients who received at least one dose of double-blind study drug, and had both a baseline assessment and at least one post-baseline assessment of any primary or secondary efficacy variable. HbA_1c_ was measured at a National Glycohemoglobin Standardization Program (NGSP) Level 1 certified central laboratory (Covance Central Laboratory Services), using a high performance liquid chromatography method.

Secondary efficacy measures included the percentage of patients reaching HbA_1c_ <7.0% or ≤6.5%, FPG, 2-h postprandial glucose (PPG) and glucose excursion (defined as 2-h PPG minus plasma glucose 30 min prior to the meal test before study drug administration), 7-point SMPG, body weight, insulin dose, patients requiring rescue therapy and safety and tolerability. The PPG measurements were assessed after intake of a standardized 600 kcal liquid test breakfast (400 ml of Ensure Plus®, Abbott Nutrition, Columbus, OH, USA; 53.8% carbohydrate, 16.7% protein and 29.5% fat; consumed within a 10-min period performed 30 min after drug administration) at baseline and week 24.

Safety and tolerability included reported adverse events and other safety information such as symptomatic hypoglycaemia (clinical symptoms of hypoglycaemia accompanied by plasma glucose <60 mg/dl [3.3 mmol/l] or associated with prompt recovery after oral carbohydrate, intravenous glucose or glucagon administration if no plasma glucose measurement was available); severe symptomatic hypoglycaemia (clinical symptoms in which the patient required assistance of another person, accompanied by plasma glucose <36 mg/dl [2.0 mmol/l] or associated with prompt recovery after oral carbohydrate, intravenous glucose or glucagon administration if no plasma glucose measurement was available); local tolerability at injection site; allergic or allergic-like reactions; suspected pancreatitis and major cardiovascular events, vital signs, 12-lead ECG and laboratory tests. The safety population comprised all randomized patients exposed to at least one dose of study drug; the on-treatment period for safety assessments was defined as the time from the first dose of double-blind study drug up to 3 days after the last dose.

### Statistical Analyses

Sample sizes of 145 patients in each of the two study groups were calculated to provide a statistical power of 90% assuming the common standard deviation of 1.3% to detect a 0.5% difference in change from baseline to week 24 in HbA_1c_ between lixisenatide and placebo. Statistical significance was assumed at the 5% level, all tests were two-sided. Analyses of the primary efficacy variable [change in HbA_1c_ from baseline to endpoint using the last observation carried forward (LOCF)] were performed using an analysis of covariance (ancova) model with treatment group, screening strata for HbA_1c_ (<8%/≥8%), sulfonylurea use (yes/no), and country as fixed factors, and baseline HbA_1c_ as a covariate. Continuous secondary efficacy variables were also analysed by ancova, and categorical secondary efficacy variables were analysed using a Cochran–Mantel–Haenszel method stratified on randomization strata. Summaries of safety data (descriptive statistics and frequency tables) were presented by treatment group. Statistical analyses were performed by using the Statistical Analysis System software version 9.2.

## Results

A total of 311 patients were randomized to one of the two treatment groups (154 lixisenatide, 157 placebo) and all received at least one dose of double-blind treatment. Patients had a mean age of 58.4 years, diabetes duration of 13.9 years, BMI of 25.3 kg/m^2^ and baseline HbA_1c_ of 8.53%. Approximately 70% of patients were receiving a sulfonylurea at screening. The mean duration of treatment with basal insulin was approximately 3 years, with around 60% of the patients receiving insulin glargine, 27% insulin detemir and 13% NPH. Demographic and baseline characteristics were well matched and there were no clinically relevant differences between the two groups (Table 1). Thirty-four patients (10.9%) discontinued prematurely from study treatment [21 (13.6%) lixisenatide, 13 (8.3%) placebo], mainly because of adverse events [14 (9.1%) lixisenatide, 5 (3.2%) placebo]. Approximately 82% of patients reached and stayed on the lixisenatide maintenance dose of 20 µg once daily at week 24. The cumulative exposure to study treatment was 65.0 and 69.4 patient-years for lixisenatide and placebo, respectively, with a median duration on treatment of 169 days in both groups.

**Table 1:** Patient disposition, demographics and baseline characteristics (safety population)

	Lixisenatide (n = 154)	Placebo (n = 157)
Age (mean ± s.d.) (years)	58.7 ± 10.2	58.0 ± 10.1
Male, n (%)	69 (44.8%)	80 (51.0%)
Race, n (%)		
Asian/Oriental	154 (100%)	157 (100%)
Japan	72 (46.8%)	87 (55.4%)
Republic of Korea	67 (43.5%)	56 (35.7%)
Philippines	13 (8.4%)	5 (3.2%)
Taiwan	2 (1.3%)	9 (5.7%)
BMI (mean ± s.d.) (kg/m^2^)	25.4 ± 3.7	25.2 ± 3.9
Duration of diabetes since diagnosis (mean ± s.d.) (years)	13.7 ± 7.7	14.1 ± 7.7
HbA_1c_ <8%, n (%)	35 (22.7%)	36 (22.9%)
HbA_1c_ ≥8%, n (%)	119 (77.3%)	121 (77.1%)
Sulfonylurea use at screening		
Yes	108 (70.1%)	111 (70.7%)
No	46 (29.9%)	46 (29.3%)
Duration of treatment with sulfonylurea (mean ± s.d.) (years)	5.33 ± 4.83	6.80 ± 5.24
Insulin use at screening		
Duration of treatment with basal insulin (mean ± s.d.) (years)	2.94 ± 3.67	3.01 ± 4.27
Total daily insulin dose, (mean ± s.d.) (U)	24.9 ± 14.0	24.1 ± 14.2
Glargine [n = 187 (60%)]	25.1 ± 13.4	23.8 ± 12.3
Detemir [n = 83 (27%)]	19.9 ± 8.7	21.2 ± 14.3
NPH [n = 39 (13%)]*	35.0 ± 20.5	28.8 ± 18.2
Premix [n = 2 (<1%)]†	0	48.0 ± 25.5
*Efficacy variables at baseline*		
HbA_1c_ (mean ± s.d.) (%)	8.54 ± 0.73	8.52 ± 0.78
FPG (mean ± s.d.) (mmol/l)	7.67 ± 2.32	7.75 ± 2.25
2-h PPG (mean ± s.d.) (mmol/l)	17.81 ± 3.36	17.75 ± 3.94
2-h glucose excursion (mean ± s.d.) (mmol/l)	9.72 ± 3.27	9.70 ± 4.19
Average 7-point SMPG (mean ± s.d.) (mmol/l)	11.58 ± 2.51	11.42 ± 2.46
Body weight (mean ± s.d.) (kg)	65.93 ± 13.00	65.60 ± 12.47

BMI, body mass index; FPG, fasting plasma glucose; HbA_1c_, glycated haemoglobin; PPG, postprandial plasma glucose; s.d., standard deviation; SMPG, self-monitored plasma glucose.

* NPH included Isophane insulin and Insulin human injection, isophane.

† Protocol deviation; Mixed insulin included Novomix.

### Efficacy

**Figure 1:**
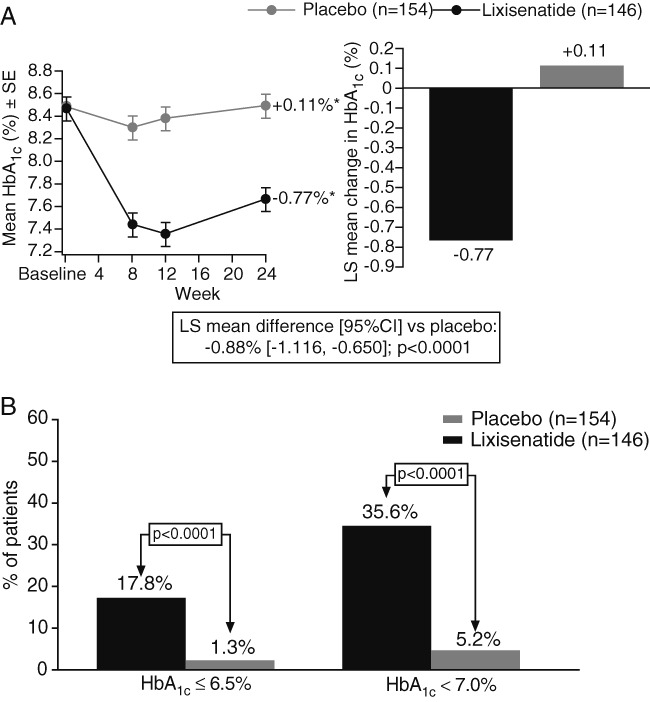
Glycated hemoglobin (HbA_1c_) levels after 24 weeks. (A) Mean (± s.e.) HbA_1c_ over time. (B) Percentage of patients achieving HbA_1c_ goals <7.0% and ≤6.5%; *LS mean change in HbA_1c_ at week 24, LOCF data. mITT population.

Mean baseline HbA_1c_ was 8.5% in both groups. The LS mean change from baseline to endpoint (week 24) was −0.77% for the lixisenatide group and +0.11% for the placebo group (LS mean difference vs. placebo: −0.88%, 95% CI = [−1.116, −0.650]; p < 0.0001) (figure 1A). HbA_1c_ in the lixisenatide group was already decreased at week 8 and remained reduced during the whole treatment period compared with the placebo group, where no relevant change in HbA_1c_ was observed (figure 1A). The goal of HbA_1c_ <7.0% and the stricter goal of HbA_1c_ ≤6.5% were both achieved by significantly more lixisenatide patients compared with placebo patients (both p < 0.0001; figure 1B).

**Figure 2:**
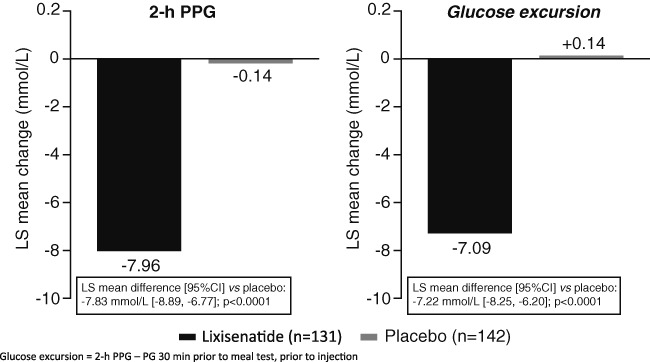
Changes in post-meal glucose parameters from baseline after 24 weeks. Change in LS mean (±s.e.) 2-h postprandial plasma glucose (PPG) levels and change in LS mean (±s.e.) 2-h glucose excursion (2-h PPG – plasma glucose 30 min prior to meal test, prior to injection). LOCF data. mITT population.

During the standardized meal test, treatment with lixisenatide significantly improved post-prandial glycaemic control as shown by the significant decrease in 2-h PPG values and blood glucose excursions from baseline to week 24 compared with the placebo group (both p < 0.0001; figure 2). Other secondary endpoints also demonstrated significant improvements in metabolic control with lixisenatide compared with placebo. Significant differences versus placebo were achieved for LS mean changes in average 7-point SMPG (−1.91 ± 0.27 mmol/l for lixisenatide vs. −0.56 ± 0.27 mmol/l for placebo; p < 0.0001), FPG (−0.42 ± 0.31 mmol/l for lixisenatide vs. +0.25 ± 0.30 mmol/l for placebo; p = 0.0187) and daily basal insulin dose (−1.39 ± 0.46 U for lixisenatide vs. −0.11 ± 0.44 U for placebo; p = 0.0019). Two (1.3%) lixisenatide patients and five (3.2%) placebo patients required rescue therapy.

Mean changes in body weight were small, but there was a trend to weight decrease with lixisenatide in this insulin-treated population, with no statistically significant differences between lixisenatide and placebo (LS mean change: −0.38 vs. +0.06 kg, respectively; 95% CI = [−0.925, 0.061]; p = 0.0857).

### Safety and Tolerability

Overall, the incidence of treatment-emergent adverse events (TEAEs) was higher in the lixisenatide group than the placebo group, mostly attributable to gastrointestinal and hypoglycaemic events (Table 2). Most of the gastrointestinal events were transient, mild to moderate in intensity and resolved spontaneously without sequelae. The frequency of serious TEAEs was similar in the two groups — 10 (6.5%) in the lixisenatide group and nine (5.7%) in the placebo group. Two patients (1.3%) in the lixisenatide group experienced TEAEs of cerebrovascular infarction, which were assessed by the CAC as non-fatal ischaemic stroke; treatment was discontinued in both patients.

**Table 2:** Number (%) of patients with treatment-emergent adverse events (TEAEs) occurring in ≥5% (preferred term) in either group, symptomatic hypoglycaemia and injection site reactions

TEAE, n (%)	Lixisenatide(n = 154)	Placebo(n = 157)
Any TEAE	137 (89.0)	110 (70.1)
Any serious TEAE	10 (6.5)	9 (5.7)
TEAE leading to death	0	1 (0.6)
Discontinuation due to a TEAE	14 (9.1)	5 (3.2)
Gastrointestinal disorders (any)	94 (61.0)	23 (14.6)
Any TEAE occurring in ≥5% of patients in either group (Preferred term)*		
Nausea	61 (39.6)	7 (4.5)
Vomiting	28 (18.2)	3 (1.9)
Nasopharyngitis	21 (13.6)	20 (12.7)
Headache	16 (10.4)	3 (1.9)
Dizziness	13 (8.4)	8 (5.1)
Abdominal discomfort	11 (7.1)	1 (0.6)
Dyspepsia	11 (7.1)	0
Asthenia	10 (6.5)	12 (7.6)
Diarrhoea	10 (6.5)	4 (2.5)
Decreased appetite	10 (6.5)	0
Constipation	8 (5.2)	4 (2.5)
Injection site reactions	2 (1.3)	2 (1.3)
Symptomatic hypoglycaemia (per protocol definition)	66 (42.9)	37 (23.6)
Blood glucose <60 mg/dL	59 (38.3)	32 (20.4)

TEAE, treatment-emergent adverse events.

* Excluding symptomatic hypoglycaemia.

More patients in the lixisenatide group [14 (9.1%)] discontinued treatment due to a TEAE compared with the placebo group [5 (3.2%)], mainly as a result of gastrointestinal TEAEs — six (3.9%) and four (2.6%) patients in the lixisenatide group discontinued treatment due to nausea and vomiting, respectively, versus none in the placebo group. One death was reported during the treatment period (a case of suicide in the placebo group).

Hypoglycaemia was the most frequently reported TEAE in the lixisenatide group — 66 (42.9%) lixisenatide-treated patients and 37 (23.6%) placebo-treated patients reported symptomatic hypoglycaemia meeting the protocol-specified definition. None of these events was severe. In the subgroup of patients not receiving a sulfonylurea, the incidence of hypoglycaemia in lixisenatide-treated patients was close to that of placebo: 32.6% versus 28.3%, respectively (Table 3).

**Table 3:** Hypoglycaemia by sulfonylurea use at screening

	Lixisenatide		Placebo	
		
Sulfonylurea use at screening	Yes (n = 108)	No (n = 46)	Yes (n = 111)	No (n = 46)
Patients with symptomatic hypoglycaemia, n (%)	51 (47.2%)	15 (32.6%)	24 (21.6%)	13 (28.3%)
Events / patient year, n	3.54	1.48	1.48	1.36
Patients with blood glucose <60 mg/dl, n (%)	46 (42.6%)	13 (28.3%)	21 (18.9%)	11 (23.9%)

No pancreatitis was reported during this study. One lixisenatide-treated patient had an increase in lipase, which was <3 ULN, and one placebo-treated patient had an increase in pancreatic enzymes with lipase ≥3 ULN. No increases in calcitonin or TEAEs related to the thyroid gland were reported.

A total of four patients (two patients in each group) experienced injection site reactions; none of the reactions were considered serious or severe or led to treatment discontinuation. A total of seven possible allergic reactions were reported (five events in the lixisenatide group and two events in the placebo group) during the on-treatment period. Only one of these events (urticaria, in a lixisenatide-treated patient) was adjudicated by the ARAC as an allergic reaction possibly related to study medication.

## Discussion

In this Phase III, randomized, placebo-controlled trial, lixisenatide 20 µg once daily as add-on to basal insulin with or without sulfonylureas met its primary endpoint of significantly improving HbA_1c_ versus placebo (between-group difference −0.88%; p < 0.0001). Significantly more lixisenatide patients achieved HbA_1c_ targets of ≤6.5% (17.8%) and <7.0% (35.6%) than placebo (1.3 and 5.2%; p < 0.0001). The magnitude of improvement in HbA_1c_ is consistent with that seen with lixisenatide monotherapy administered once daily for 12 weeks in previously drug-naïve patients with type 2 diabetes [10] and as add on to metformin in a 13-week study [11] in a primarily Caucasian population.

The only other published prospective randomized controlled trial looking at addition of a GLP-1 receptor agonist to insulin therapy compared exenatide twice daily and placebo in 259 patients on insulin glargine (with or without metformin and/or pioglitazone) [24]. After 30 weeks, HbA_1c_ decreased by 1.7% (baseline 8.4%) with exenatide twice daily and 1.0% (baseline 8.5%) with placebo – a significant between-group difference of −0.7%. It should be noted, however, that the study population in this exenatide trial was predominantly Caucasian, with only 3% of patients being of Asian origin.

Although several studies have looked at the efficacy and safety of other GLP-1 receptor agonists (either as monotherapy or add-on to oral agents) in Japanese populations [14–16,18–22], comparisons with the present lixisenatide study need to be interpreted with caution due the different background therapies and the additional inclusion of non-Japanese (predominantly Korean) patients in our study. Furthermore, the difference in the population has to be taken into account as, in this study with basal insulin as background therapy, the diabetes duration (lixisenatide 13.7 years and placebo 14.1 years) was longer compared with other trials, indicating a population at a more advanced stage of diabetes disease. In a purely Japanese population, the maximum liraglutide dose of 0.9 mg once daily given as monotherapy reduced HbA_1c_ after 24 weeks by 1.9% (baseline 8.8%) versus a reduction of 1.4% with glibenclamide 2.5 mg/day [16]. At Week 52, after an open-label extension period, the mean HbA_1c_ reduction relative to glibenclamide was 0.49% [20]. In another 24-week study, liraglutide 0.9 mg once daily as add-on to sulfonylurea monotherapy reduced HbA_1c_ by 1.6% (baseline 8.2%; placebo-subtracted: −1.3%) without causing any major hypoglycaemic episodes, although higher rates of minor hypoglycaemia were reported among subjects in the liraglutide group than in those on placebo [21]. At week 52, after an open-label extension period, the mean placebo-subtracted reduction in HbA_1c_ was 1.33% [15]. Data are also available for exenatide as an add-on to sulfonylurea-based oral mono-or combination in a purely Japanese population. After 24 weeks, the maximum exenatide dose of 10 µg twice daily provided an HbA_1c_ reduction of 1.6% (baseline 8.2%) versus a reduction of 0.3% (baseline 8.1%) with placebo, with mild-to-moderate hypoglycaemia reported in 58 and 23% of patients, respectively [18]. Efficacy was well sustained during 28 weeks of open-label extension [22].

Data from populations with a significant proportion of Korean patients are rare. The study by Yang and colleagues, which included 18% Korean patients (the remainder were of Chinese or Indian origin), looked at liraglutide add-on to metformin over 16 weeks and yielded results similar to the global liraglutide Phase III trials [23]. In the study by Gao and co-workers, 17% of patients were of Korean descent (the rest were of Chinese, India and Taiwanese descent) – exenatide add-on to metformin (with or without a sulfonylurea) over 16 weeks had an efficacy/safety profile consistent with that seen in non-Asian patients [17].

Lixisenatide had a pronounced effect on postprandial glycaemic control, significantly improving 2-h PPG and glucose excursion. These results are also consistent with reports from previous studies of lixisenatide [9–12]. The PPG effect of lixisenatide (2-h glucose excursion −7.22 mmol/l vs. placebo; p < 0.0001) appears to be greater than that seen with addition of exenatide twice daily to insulin therapy (albeit in a predominantly Caucasian population) [24] – morning 2-h glucose excursion (but based on SMBG profiles, rather than during a meal test) was −2.0 mmol/l with exenatide twice daily versus −0.2 mmol/l with placebo (between-group difference: −1.8 mmol/l; p < 0.001).

Furthermore, PPG makes a greater contribution to HbA_1c_ as patients start to approach recommended HbA_1c_ goals (at HbA_1c_ >8.5%, FPG makes the predominant contribution to overall glycaemic control, whereas PPG becomes more relevant at lower HbA_1c_ levels).Targeting FPG with basal insulin in patients insufficiently controlled (HbA_1c_ <7%) on oral agents has been shown to markedly increase the relative contribution of PPG to overall glycaemia from 20–24 to 59–69% [25]. Thus, in terms of achieving HbA_1c_ targets, a focus on PPG may become increasingly relevant at lower (but still suboptimal) HbA_1c_ levels [26], and PPG may be associated with diabetes-related complications both independently and through a contribution to overall glycaemia [27]. Accordingly, guidelines recommend targeting PPG, FPG and HbA_1c_ simultaneously for treatment of type 2 diabetes; in terms of PPG, the International Diabetes Federation (IDF) considers a 2-h PPG target <7.8 mmol/l (140 mg/dl) to be both “reasonable and achievable” [27].

Lixisenatide also significantly improved FPG and average 7-point SMBG levels relative to placebo. When interpreting these plasma glucose results, it should be noted that there was no formal titration of basal insulin doses and the protocol specified that the dose was to be kept stable within a ±20% range. Nevertheless, a significant reduction in the daily basal insulin dose was seen with lixisenatide compared with placebo.

A statistically significant decrease in weight with lixisenatide versus placebo has been reported previously [11]. In the present study, typical of an Asian population with relatively low mean baseline BMI and body weight (25 kg/m^2^ and 66 kg, respectively), observed weight changes were generally small. Nevertheless, maintaining weight stability represents an important achievement in an insulin-treated population, who typically would be expected to gain weight, especially with concomitant sulfonylurea therapy. Despite the small changes, there was a clear trend towards weight loss for lixisenatide compared with placebo (p = 0.0857).

Lixisenatide was generally well tolerated in the present study. Overall, 86.4% of patients in the lixisenatide group completed the study compared with 91.7% on placebo, and 81.8% of patients were still on full doses of lixisenatide at the end of the double-blind treatment period. As expected, the most frequent adverse events were gastrointestinal in nature – mainly nausea, with lower rates of vomiting and other gastrointestinal symptoms. The frequency of nausea (39.6% for lixisenatide) is slightly higher in this exclusively Asian population than that observed with lixisenatide monotherapy or add-on to metformin at the 20 µg once daily dose (22–25%) in a global population [10,11].

Hypoglycaemia was not unexpected in this insulin-treated population, particularly considering that 70% were also receiving sulfonylureas. A higher incidence was reported in those lixisenatide-treated patients who were also receiving sulfonylureas, while the incidence in those receiving purely basal insulin in combination with lixisenatide was close to the rate with placebo. There were no reports of severe hypoglycaemia.

In conclusion, lixisenatide administered once daily as an add-on treatment to basal insulin with or without a sulfonylurea in Asian patients with type 2 diabetes and FPG at screening <250 mg/dl provided a significant improvement in HbA_1c_ and a pronounced effect on postprandial glucose control. Overall, lixisenatide was well tolerated in this population. These results support those of other Phase III studies [28–30], highlighting the potential of lixisenatide for further development as a glucose-lowering agent to treat patients with type 2 diabetes.

## Appendix

### Principal Investigators for the EFC10887 (GETGOAL-L Asia) Study Group

*Japan*: Nobuyuki Abe, Keiko Arai, Tsuguyoshi Asano, Atsushi Hasegawa, Toru Hiyoshi, Toshihiko Inoue, Yukinori Isomura, Sizuka Kaneko, Tadashu Kasahara, Zenji Makita, Kiyokazu Matoba, Hiroaki Miyaoka, Tetsuji Niiya, Keiichiro Nishino, Katsumi Noda, Akira Okada, Yukiko Onishi, Takeshi Osonoi, Mitsuru Ozaki, Masatomo Sekiguchi, Toshihiko Shiraiwa, Hidekatsu Sugimoto, Yoshihiko Suzuki, Toru Takeuchi, Tsuyoshi Tanaka, Miki Tateyama, Osamu Tomonaga, Hiroshi Uchino, Nobuaki Watanabe, Shuichi Watanabe, Takayuki Watanabe, Akira Yamauchi, Tatsuo Yanagawa.

*Philippines*: Maria Honolina Gomez, Araceli Panelo, Rosa Allyngsy, Ernesto L Ang.

*Republic of Korea*: Hong-Sun Baek, H. Choon Chung, Hak∼C. Jang, Dong-Jun Kim, In J. Kim, Kwang-Won Kim, Yong S. Kim, Hyun Chul Lee, Ji Hyun Lee, Kwan-Woo Lee, Kyung Wan Min, Chul Woo Anh, Doo Man Kim, Ie B. Park, Minho Shong, Young D. Song, Hyun Shik Son, Ki-Ho Song, Kyu C. Won, Jae M. Yu.

*Taiwan*: Wayne H Sheu, Dee Pei, Chwen-Tzuei Chang.
